# Differences and allometric relationships among assimilative branch traits of four shrubs in Central Asia

**DOI:** 10.3389/fpls.2022.1064504

**Published:** 2022-12-13

**Authors:** Huan-Huan Meng, Ben-Feng Yin, Yong-Gang Li, Xiao-Bing Zhou, Yuan-Ming Zhang, Ye Tao, Duo-Qi Zhou

**Affiliations:** ^1^ Anhui Province Key Laboratory of the Biodiversity Study and Ecology Conservation in Southwest Anhui, College of Life Sciences, Anqing Normal University, Anqing, Anhui, China; ^2^ State Key Laboratory of Desert and Oasis Ecology, Xinjiang Institute of Ecology and Geography, Chinese Academy of Sciences, Urumqi, Xinjiang, China

**Keywords:** allometry, desert shrubs, ecological adaptability, convergent adaptation, assimilative branches

## Abstract

Shrubs play a major role in maintaining ecosystem stability in the arid deserts of Central Asia. During the long-term adaptation to extreme arid environments, shrubs have developed special assimilative branches that replace leaves for photosynthesis. In this study, four dominant shrubs with assimilative branches, namely *Haloxylon ammodendron*, *Haloxylon persicum*, *Calligonum mongolicum*, and *Ephedra przewalskii*, were selected as the research objects, and the dry mass, total length, node number, and basal diameter of their assimilative branches and the average length of the first three nodes were carefully measured, and the allometric relationships among five traits of four species were systematically compared. The results indicated that: (1) Four desert shrubs have different assimilative branches traits. Compared with *H. persicum* and *H. ammodendron*, *C. mongolicum* and *E. przewalskii* have longer internodes and fewer nodes. The dry mass of *H. ammodendron* and the basal diameter of *H. persicum* were the smallest; (2) Significant allometric scaling relationships were found between dry mass, total length, basal diameter, and each trait of assimilative branches, all of which were significantly less than 1; (3) The scaling exponents of the allometric relationship between four traits and the dry mass of assimilative branches of *H. persicum* were greater or significantly greater than those of *H. ammodendron*. The scaling exponents of the relationships between the basal diameter, dry mass, and total length of *E. przewalskii* were higher than those of the other three shrubs. Therefore, although different species have adapted to drought and high temperatures by convergence, there was great variability in morphological characteristics of assimilative branches, as well as in the scaling exponents of relationships among traits. The results of this study will provide valuable insights into the ecological functions of assimilative branches and survival strategies of these shrubs to cope with aridity and drought in desert environments.

## Introduction

The environment profoundly affects the creation and variation of plant morphological structure. The environmental conditions in the arid region in China, Central Asia are harsh, and plants have developed various adaptation strategies under adverse conditions such as high temperature, drought, and soil nutrient deficiency for a long time (Gonzalez-Paleo and Ravetta, 2018; Li et al., 2018; Brodribb et al., 2020; Peguero-Pina et al., 2020; Yao et al., 2021; Zhou et al., 2022). As the organs with plasticity in the process of plant evolution, photosynthetic systems (e.g., leaves) are very sensitive to changes in environmental factors such as water, temperature, light, and CO_2_ concentration (Gratani et al., 2006; Bradshaw, 2006), and their traits are important indicators of plant adaptation strategies in response to environmental changes (Wei et al., 2020; Bergholz et al., 2021; Zhou et al., 2022).

The shrubs with assimilative branches, e.g., *Haloxylon ammodendron*, *Haloxylon persicum*, *Calligonum mongolicum*, and *Ephedra przewalskii*, are typical xerophyte species, which are widely distributed in desert regions of Central Asia (Orlovsky and Birnbaum, 2002; Hu et al., 2021). During the long-term adaptation to the adverse conditions of frequent drought and high temperature (Zhou et al., 2022), the leaves of the four shrubs degenerated into membrane scales and fleshy specialization or completely degenerated and shrunk to thin rods, and the annual woodless branches replaced the leaves for photosynthesis, completing the life history of plants (Pyankov et al., 1999; Li et al., 2011; Liu et al., 2016). They can resist various stresses, such as sterility, drought, high temperature, salt, and sand burial, and play an important role in maintaining the structure and function of desert ecosystems, reducing wind speed, improving desert microclimate, and promoting the establishment and growth of other desert plants (Wang et al., 2011; Buras et al., 2012; Fan et al., 2016; Hu et al., 2021; Wu et al., 2021).

Composed of multiple nodes and internodes and situated at the front of the shrub branch, assimilative branches are the most vigorous parts of the four shrub branching systems (Li and Li, 1981; Zhou et al., 2022). As a consequence of long-term adaptation to arid conditions, assimilative branches help the plants reduce heat load and water loss, and their vertical growth directly reduces the amount of radiation absorbed (Ehleringer and Cooper, 1992; Li et al., 2014; Ávila-Lovera et al., 2020). In addition, when the assimilative branches experience extreme drought, their surface area can be reduced by curling, wilting, or falling off to reduce water loss (Yu et al., 2012). The formation of assimilative branches is the culmination of the evolution of vegetation under drought conditions (Lyshede, 1979). Studies have shown that plants with assimilative branches are better able to adapt to drought conditions than other plants (Delzon et al., 2010; Li et al., 2014; Zhou et al., 2022). Assimilative branches are situated between leaves and stems and have unique structural characteristics, and their traits affect the carbon sequestration, competitive ability, and mechanical stability of the plant (Li and Li, 1981; Shuang et al., 2009; Li ZB et al., 2013; Yue et al., 2013; Zhou et al., 2022).

Studies have shown that plants eventually develop a combination of traits to adapt to the environment through the internal adaptation of different functional traits, and there is a certain correlation among the different trait combinations (Xue and Cao, 2010; Reich and Cornelissen, 2014). Allometric scaling is a phenomenon that reflects the link between different relative growth rates and both the two traits of an organism. Since first proposed, the proportional relationship among plant functional traits has been proved to be a valid theory that is also widely used (West et al., 1997; Niklas and Enquist, 2001; Niklas, 2004; Han and Fang, 2008; Wu et al., 2021). Studies have shown that the scaling exponents of the relationships between leaf dry mass, twig mass, and leaf area are isometric (Liu et al., 2008). At the whole plant level, the proportionate allocation of biomass in leaves versus stems, supporting the leaves scale, is in accordance with the 3/4 power law expected for the metabolic theory (Huang et al., 2016). Desert plants have internal coordination among different functional traits to cope with heat and drought stresses by making morphological adjustment, which is the most intuitive strategy (Li and Xu, 2008), and a series of morphological traits of assimilative branches are critical for plant survival in arid environments. Researchers discovered significant allometric relationships between *H. ammodendron* twig mass, dry mass fraction of assimilative branches, and stem mass, as well as the surface area of assimilative branches and stem cross-sectional area (Li ZB et al., 2013). For decades, the morphological and anatomical structures, as well as the photosynthetic physiological characteristics, of green assimilative branches have been investigated (Li and Li, 1981; Su et al., 2005; Yan et al., 2008; Liu et al., 2016; Zhang et al., 2018; Wang et al., 2021; Zhang et al., 2022). However, very limited information is available on the convergence or divergence of morphological traits of assimilative branches of various desert shrubs.

In the present study, four dominant desert shrubs, including *H. ammodendron*, *H. persicum*, *C. mongolicum*, and *E. Przewalskii* in the Gurbantunggut Desert, northwestern China, were selected as the research objects, and the total length of assimilative branches, the total number of nodes, the basal diameter, the dry mass, and other traits were determined. The purpose of this study was to explore the differences in morphological traits of assimilative branches among different shrubs and the allometric relationships between these traits. Although different species have adapted to drought and high temperature by convergence, according to existing studies, we hypothesize that different shrub species have different functional traits and trait associations of assimilative branches. The results of this study will provide valuable insights into the ecological functions of assimilative branches and the survival strategies of these shrubs to adapt to arid desert environments.

## Materials and methods

### Study site

The study site is located in the southeast part (44.34° N, 87.85° E, altitude of 515 m) of the Gurbantunggut Desert (34°09′–49°08′N, 73°25′–96°24′E), northern Xinjiang, China. This desert (4.88 × 10^4^ km^2^) is the second largest desert in China and also a typical temperate inland desert in Central Asia. It is characterized as an area with low precipitation of 50–200 mm, mean relative humidity of < 45%, high evaporation rate (> 2000 mm), long winter, short spring, sufficient sunshine, and wide daily and annual temperature ranges (Zhang and Chen, 2002; Wang et al., 2003; Tao et al., 2013). The Gurbantunggut Desert is China’s only fixed semi-fixed desert that is affected by cold and humid air currents in the Atlantic Ocean (Qian and Wu, 2010). As a result, the climate, vegetation composition, and richness in the Gurbantunggut Desert vary similarly to that of any other desert in China. (Zhang and Chen, 2002). The desert vegetation is mainly composed of *H. persicum*, *H. ammodendron*, and small shrubs such as *E. przewalskii* and *Artemisia songarica*, as well as ephemeral and ephemeroid plants that do not exist in other temperate deserts in China (Mao and Zhang, 1994; Wang et al., 2003; Yuan and Tang, 2010). In addition, biological soil crusts are widely distributed, and they are important biological factors that maintain the stability of the surface of deserts (Mao and Zhang, 1994; Zhang and Chen, 2002; Zhang and Wang, 2008).

### Sample collection

**Figure 1:**
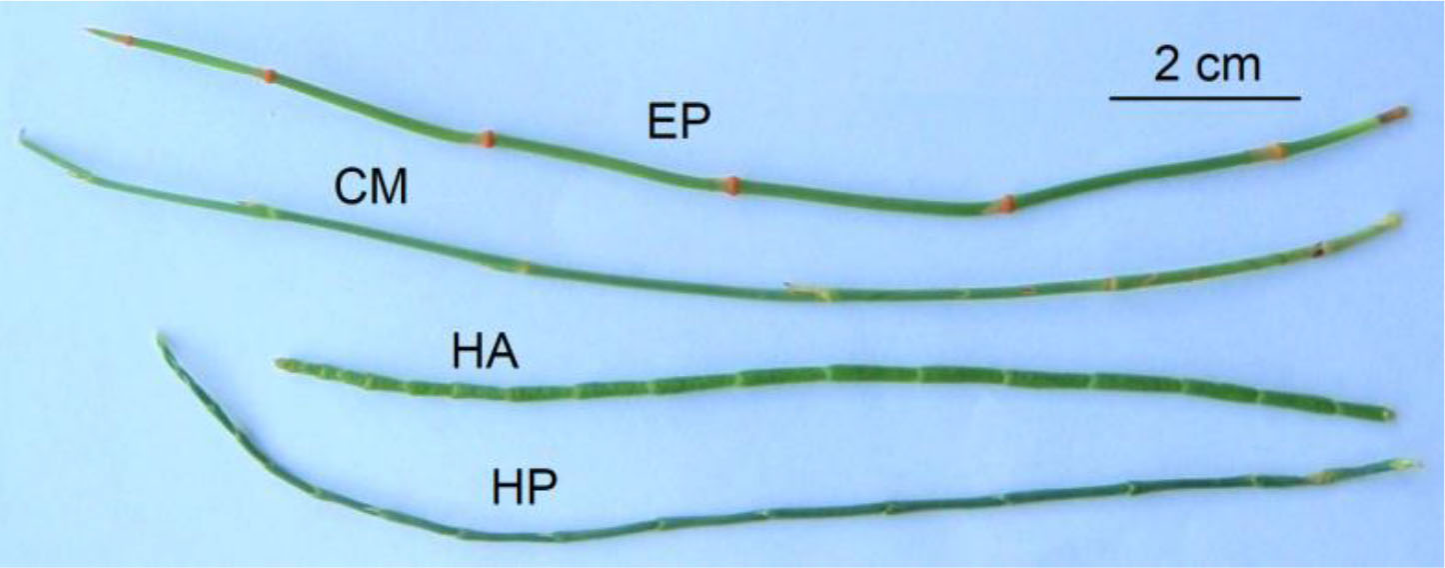
The morphology of assimilative branches of four shrubs (EP: *E. przewalskii*; CM: *C. mongolicum*; HA: *H. ammodendron*; and HP: *H. persicum*) in the Gurbantunggut Desert.

The assimilative branches of *H. persicum*, *H. ammodendron*, *C. mongolicum*, and *E. przewalskii* (**Figure 1**) were collected from a long-term monitoring plot (Beishawo Plot) in later July 2019. Referring to the manual of methodological standards of Cornelissen et al., 2003, six well-grown individuals of *H. persicum*, *H. ammodendron*, and *C. mongolicum* species with similar height and crown size were chosen from a 100 m × 100 m sampling plot, and 10 green, mature, and fully-developed intact current-year assimilative branches from the mid and upper parts of an individual plant canopy were randomly collected in various directions for different species. For *E. przewalskii*, with a small body size, 30 plants of similar height and crown size were selected, and two intact current-year assimilative branches were collected from each individual. All plant samples (*n* = 60 for each species) were labeled and quickly placed into foam boxes with ice blocks before transferring them to the lab for analysis.

### Trait measurement

In the laboratory, the number of internodes of assimilative branches (NI) was counted, and the length of assimilative branches (LAB, cm) and also the average length of the first three internodes (i.e., located at the base, not the top) of assimilative branches (AL, cm) were measured with a ruler with the precision of 1 mm. The basal diameter of assimilative branches (BD, mm) was measured three times with a vernier caliper with the precision of 0.01 mm, and then the average BD was used. Finally, the assimilative branches were oven-dried at 70°C for 48 h to reach the constant weight, and then their dry mass (DM, g) was recorded with the scale with an accuracy of 0.1 mg. The parameters were expressed as mean ± standard error (mean ± SE).

### Statistical analysis

Based on the decomposition of Type I sums of squares, the nested ANOVA was used to separately analyze the effects of different species, individuals, and assimilative branches on all traits (Nested Procedure, SAS version 8.0; SAS Institute Inc., Cary, NC, USA) (Liu et al., 2010). One-way ANOVA was used to compare the significance of differences in morphological traits of assimilative branches of the four desert plants. Levene’s test was used to test the equality of variances, and Tukey’s HSD test was used for multiple comparisons when group variances were homogeneous; Tamhane’s T_2_ test was used for multiple comparisons of means with unequal variances (Búrquez et al., 2010). All parameters were expressed as mean ± standard error (mean ± Er). SPSS 19.0 (SPSS Inc. Chicago, Illinois, USA) was used for data analysis.

Model II regression analysis was employed to estimate the correlation between the traits of assimilative branches. The relationship between any two traits is represented by *Y* = *β*·*X^α^*, where *X* and *Y* denote two parameter values, and *α* is the scaling exponent (West et al., 1997; Niklas, 2004; Niklas, 2005; Warton et al., 2006; Han and Fang, 2008; Smith, 2009). *α* = 1 indicates an isometric relationship, i.e., the dependent variable and the independent variable change uniformly or in the same proportion; *α* ≠ 1 represents an allometric relationship (Zhu et al., 2011). When determining allometric parameters, the power function is generally converted into the equation as follows: log*Y* = log*β* + *α*· log*X*; the reduced major axis (RMA) linear regression (i.e., Model Type II) was conducted to estimate the scaling slope, a 95% confidence interval (95% *CI*), and the coefficient of determination (*R* ^2^). Allometric scaling analysis was performed using the SMATR package (Falster, 2003). Origin 2019 (Originlab Corporation, Northampton, MA, USA) was used for the graphical representation of data.

## Results

**Figure 2:**
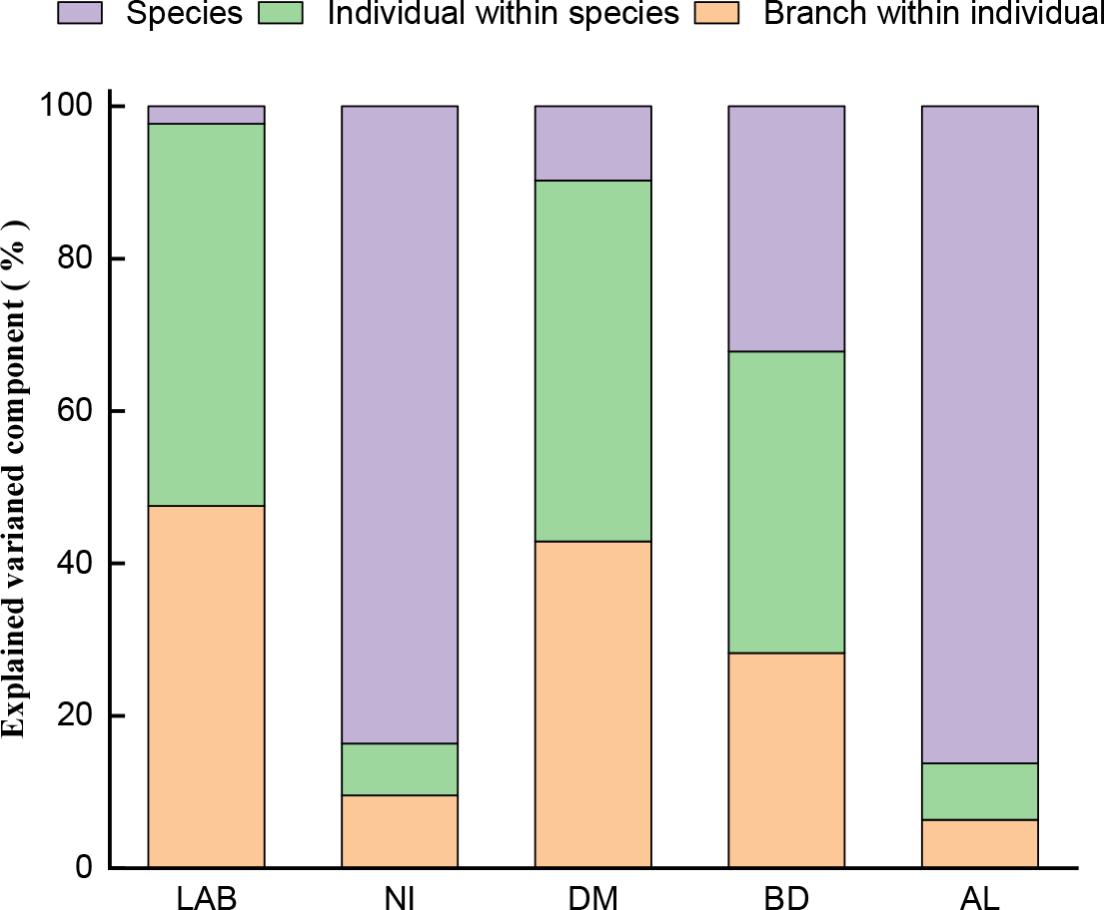
The hierarchical variation component analysis of each variable (sums of squares of type I ANOVA); LAB: Length of assimilative branches, NI: Number of internodes, DM: Dry mass of assimilative branches, BD: Average basal diameter of assimilative branches, and AL: Average length of the first three internodes of assimilating branches.

The results of the hierarchical variation component analysis of each variable (**Figure 2**) revealed that NI and AL had the most variation among species, accounting for 83.64% and 86.25% of the total variation, respectively, and little variation among individual species and assimilative branches, all of which accounted for less than 10% of the total variation. The variation among LAB and DM species was very small, accounting for less than 10% of the total variation, whereas the variation among individual species and assimilative branches was large and similar, accounting for 42.8-50.1% of the total variation. BD revealed significant variation between species, individuals, and assimilative branches, accounting for 32.2%, 39.6% and 28.2% of total variation, respectively.

### Differences in assimilative branch traits of four shrubs

**Figure 3:**
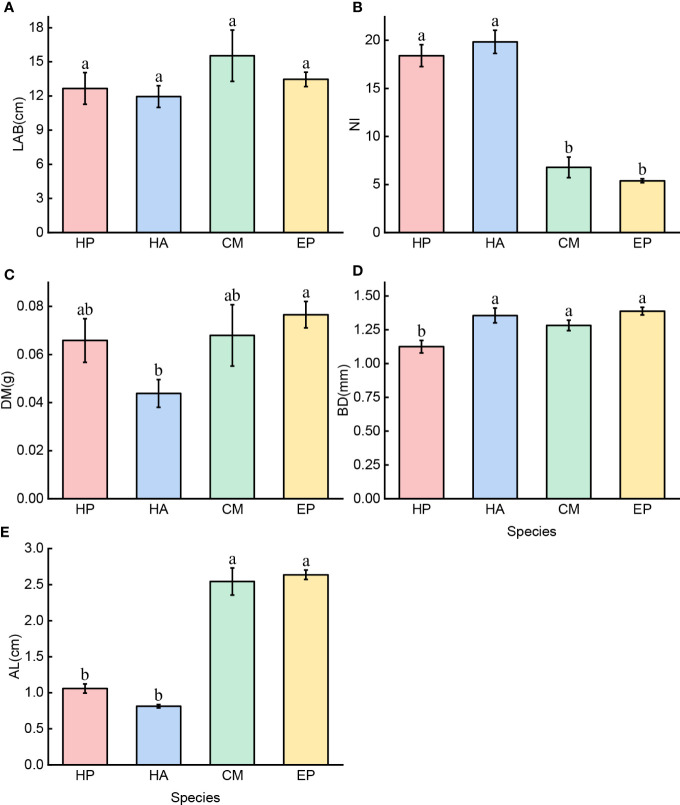
Five traits of assimilative branches of four desert shrubs; Species: HP = *Haloxylon persicum*, HA = *Haloxylon ammodendron*, CM = *Calligonum mongolicum*, and EP = *Ephedra przewalskii*; Functional traits: **(A)** LAB = Length of assimilative branches, **(B)** NI = Number of internodes, **(C)** DM = Dry mass of assimilative branches, **(D)** BD = Average basal diameter of assimilative branches, and **(E)** AL = Average length of the first three internodes of assimilative branches. Different lowercase letters represent significant differences (P < 0.05). Mean ± SE (n = 6 and 30).

*C. mongolicum* had a higher LAB (15.53 cm ± 2.26) than the other three species (13.46 cm ± 0.63, 12.66 cm ± 1.40, and 11.94 cm ± 0.95) (*P* > 0.05). (**Figure 3A**). Among the four species, *H. ammodendron* and *H. persicum* had the highest NI (19.8 ± 0.93 and 18.4 ± 1.14, respectively), while *C. mongolicum* and *E. przewalskii* had much lower NIs (6.8 ± 1.07 and 5.4 ± 0.20, respectively), and *E. przewalskii* had the lowest value (5.4 ± 0.20). (**Figure 3B**). The DM of *H. ammodendron* (0.0437* g* ± 0.006) was significantly lower than that of *E. przewalskii*, *C. mongolicum*, and *H. persicum* (0.0766* g* ± 0.006, 0.0680 g ± 0.013, and 0.0659 g ± 0.009, respectively), and no significant difference was found between the latter three species (**Figure 3C**). *E. przewalskii*, *H. ammodendron*, and *C. mongolicum* had the highest BD (1.39 mm ± 0.029, 1.36 mm ± 0.055, and 1.28 mm ± 0.038, respectively), with no significant difference between the three species; however, *H. persicum* had the lowest BD (1.13 mm ± 0.047). (**Figure 3D**). *C. mongolicum* and *E. przewalskii* had higher ALs (2.54 cm ± 0.19 and 2.64 cm ± 0.06, respectively) than the two *Haloxylon* species (1.06 cm ± 0.06 and 0.81 cm ± 0.02). (**Figure 3E**). In short, the assimilative branch traits of four desert shrubs differed.

### Allometric relationships among assimilative branch traits of four shrubs

**Figure 4:**
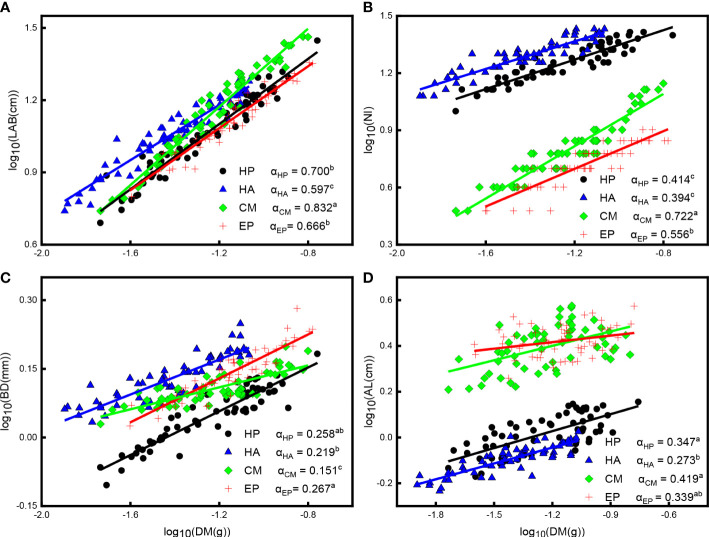
Allometric relationships among LAB, NI, BD, AL, and DM of four desert shrubs. **(A)** The relationship between LAB and DM, **(B)** The relationship between NI and DM, **(C)** The relationship between BD and DM, **(D)** The relationship between AL and DM. Species: HP = *Haloxylon persicum*, HA = *Haloxylon ammodendron*, CM = *Calligonum mongolicum*, and EP = *Ephedra przewalskii*; Functional traits: LAB = Length of assimilative branches, NI = Number of internodes, DM = Dry mass of assimilative branches, BD = Average basal diameter of assimilative branches, and AL = Average length of the first three internodes of assimilative branches; Different lowercase letters in the upper right-hand corner of the plot of scaling exponents (α) represent significant differences (P < 0.05).

There were significant positive correlations between LAB, NI, BD, and DM (*P* < 0.001), AL and DM (*P* < 0.05), and all scaling exponents were significantly less than one *P *< 0.001 (**Figure 4**; **Supplementary Table S1**), indicating that the rate of linear increase in LAB, NI, BD, and AL was lower than in DM. The scaling exponent for LAB and DM of *C. mongolicum* had the largest value (0.832), while that of *H. ammodendron* had the smallest value (0.597); the scaling exponent of *H. persicum* and *E. przewalskii* exhibited moderate values, and both these species significantly differed from the other two species (**Figure 4A**; **Supplementary Table S1**). Furthermore, for LAB vs. DM, *H. persicum* had a higher mean Y-intercept than *E. przewalskii*, indicating that *H. persicum* had a larger LAB than *E. przewalskii* at given SM. The scaling exponent between NI and DM of *C. mongolicum* showed the largest value (0.772), followed by that of *E. przewalskii* (0.556), and those of *H. persicum* and *H. ammodendron* had the smallest values with no significant difference. The Y-intercept of *H. ammodendron* was significantly higher than that of *H. persicum* (**Figure 4B**; **Supplementary Table S1**), i.e., the former species produced higher NI than the latter at a given DM. The scaling exponents between BD and DM of *E. przewalskii* and *H. persicum* (0.267 and 0.258, respectively) had considerably higher values than those of *C. mongolicum* (0.219) and *H. ammodendron* (0.151), and *H. ammodendron* had the lowest value of scaling exponent (**Figure 4C**; **Supplementary Table S1**). In addition, the Y-intercept for BD vs. DM of *E. przewalskii* was higher than that of *H. persicum*. For AL vs. DM, the scaling exponent of *C. mongolicum* and *H. persicum* had markedly higher values (0.419 and 0.347, respectively) than that of *H. ammodendron* (0.273), while *E. przewalskii* showed no significant difference in scaling slope with the other three species (**Figure 4D**; **Supplementary Table S1**).

**Figure 5:**
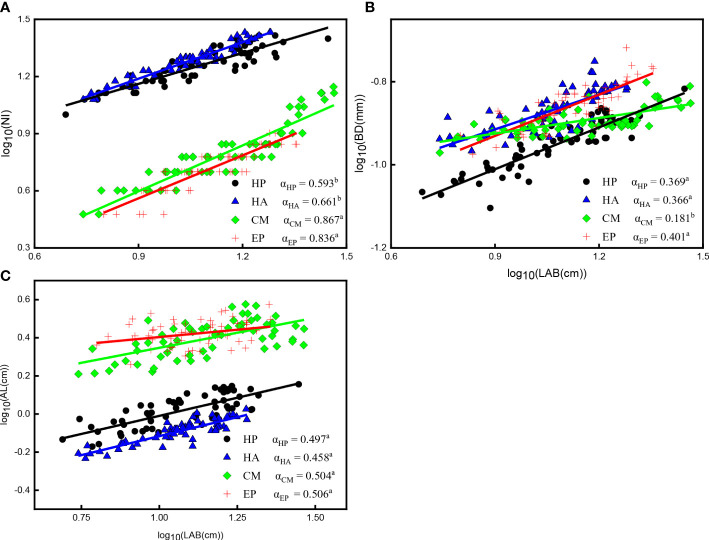
Allometric relationships among NI, BD, AL, and LAB of four desert shrubs. **(A)** The relationship between NI and LAB, **(B)** The relationship between BD and LAB, **(C)** The relationship between AL and LAB. Species: HP = *Haloxylon persicum*, HA = *Haloxylon ammodendron*, CM = *Calligonum mongolicum*, and EP = *Ephedra przewalskii*; Functional traits: LAB = Length of assimilative branches, NI = Number of internodes, BD = Average basal diameter of assimilative branches, AL = Average length of the first three internodes of assimilative branches; Different lowercase letters in the upper right-hand corner of the plot of scaling exponents (α) represent significant differences (P < 0.05).

Similarly, significant positive correlations between NI, BD, AL, and LAB of the four shrubs (*P* < 0.001) were identified (**Figure 5**; **Supplementary Table S2**). The scaling slope between NI and LAB of *C. mongolicum* (0.867) and *E. przewalskii* (0.836) did not significantly differ, but the Y-intercept of *C. mongolicum* was higher than that of *E. przewalskii*; The scaling exponents between NI and LAB of *H. persicum* and *H. ammodendron* had markedly lower values (0.661 and 0.593, respectively) than those of the other two species, and their Y-intercepts also differed (**Figure 5A**; **Supplementary Table S2**). The scaling exponents between BD and LAB of *E. przewalskii*, *H. persicum*, and *H. ammodendron* exhibited significantly higher values (0.401, 0.369, and 0.366, respectively) than that of *C. mongolicum* (0.181), and there was no significant difference in Y-intercepts between *H. ammodendron* and *E. przewalskii*, which were greater than that of *H. persicum* (**Figure 5B**; **Supplementary Table S2**). No significant differences in values of the scaling exponent between AL and LAB of the four shrubs were observed, but the Y-intercepts had significant differences among these shrubs (**Figure 5C**; **Supplementary Table S2**).

**Figure 6:**
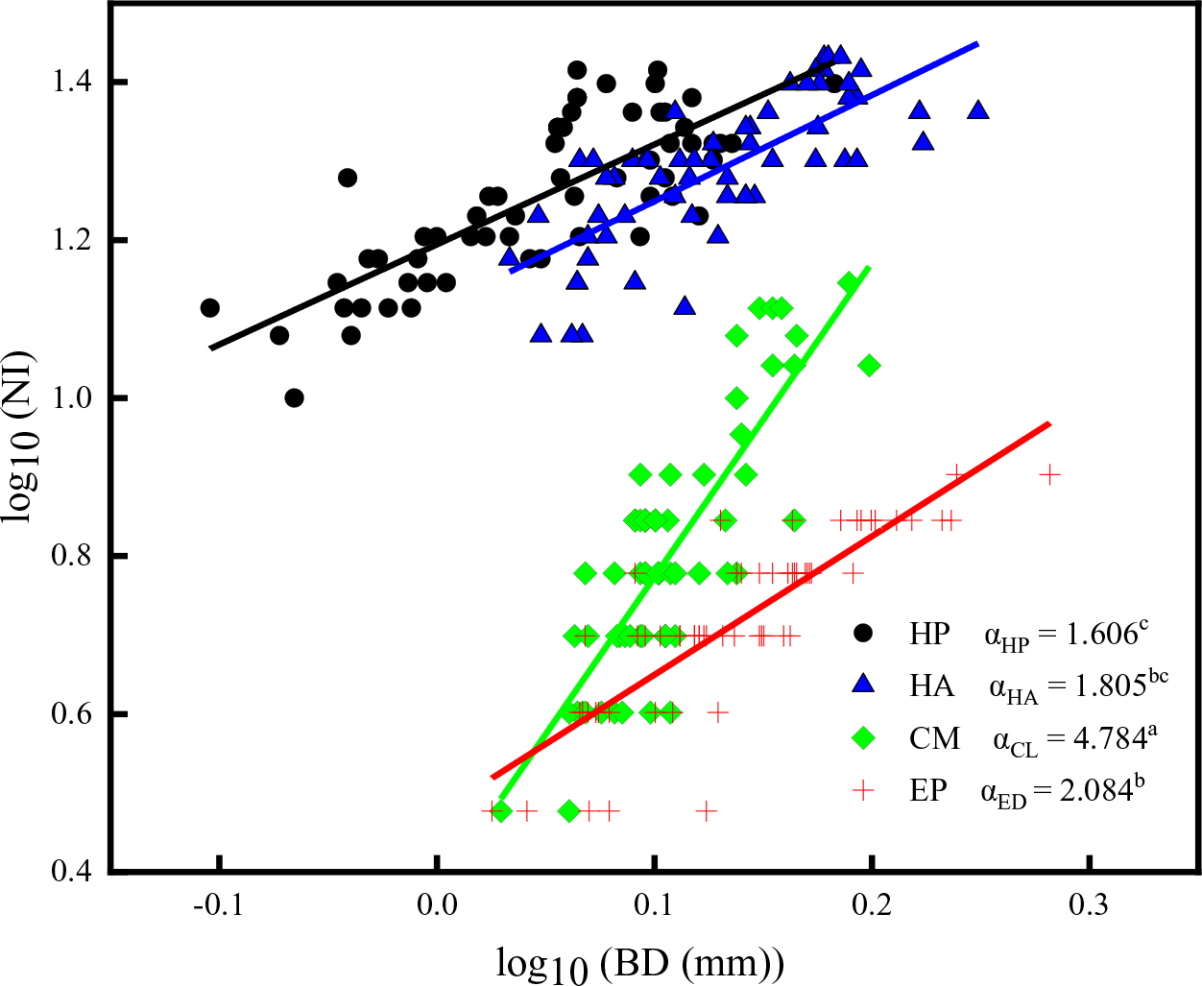
The allometric relationship between NI and BD of four desert shrubs; Species: HP = *Haloxylon persicum*, HA = *Haloxylon ammodendron*, CM = *Calligonum mongolicum*, and EP = *Ephedra przewalskii*; NI = Number of internodes, BD = Average basal diameter of assimilative branches. Different lowercase letters in the upper right-hand corner of the plot of scaling exponents (α) represent significant differences (P < 0.05).

The scaling exponents between NI and BD of the four shrubs had significantly greater values than 1 (*P* < 0.001). *C. mongolicum* had the highest scaling slope (4.784), followed by *E. przewalskii* (2.084), whereas *H. persicum* had the lowest scaling slope (1.606). There was no significant difference in scaling exponents among *E. przewalskii*, *H. ammodendron*, and *H. persicum*. The Y-intercept of *H. persicum* was significantly higher than that of *H. ammodendron*, which was also significantly higher than that of *E. przewalskii* (**Figure 6**; **Supplementary Table S3**). Consequently, the allometric relationships among multiple assimilative branch traits also showed strong inconsistency.

## Discussion

### Morphological characteristics of assimilative branches of desert shrub

Leaf morphological traits clearly reflected the response and adaptation of plants to the environment and affected the material and energy exchanges between plants and the surrounding environment, as well as the viability, development, and change in the growth direction of plants (Smith and Geller, 1980; Lambers et al., 2008; Vogel, 2009; Li et al., 2012; Jin et al., 2019; Ren, 2022). Some studies have reported that after long-term drought stress, shrubs have evolved assimilative branches to adapt to the desert environment (Li et al., 2014; Ávila-Lovera et al., 2020). The nested ANOVAs of the present study demonstrated that the variation of NI and AL was the greatest among species, whereas the variation of LAB, BD and DM was primarily affected by the plants in the sample plot and relevant to assimilative branch collection, rather than the species.

Although *H. ammodendron* and *H. persicum* are members of the same genus, the traits of their assimilative branches differed. **Figure 3** shows that *H. ammodendron* developed assimilative branches with shorter internodes, larger basal diameter, a greater number of nodes, and a lower dry mass than *H. persicum*. This morphological difference could indicate that *H. ammodendron* and *H. persicum* have evolved distinct strategies for maintaining effective photosynthesis, reducing water evaporation, and even reproducing offspring. Our findings are consistent with Zhang’s findings on the individual morphology of *H. ammodendron* and *H. persicum* (Zhang, 2002). Plants can spatially arrange their canopy structure by changing the length, diameter, and distribution of their branches, according to studies. *H. ammodendron* was larger and had greater branching ability and a denser canopy than *H. persicum* (Li et al., 2009; Wang et al., 2011; Xu et al., 2012). Therefore, we speculate that the morphological characteristics of assimilative branches of two *Haloxylon* species are related to the size and configuration of individual plants. The shorter the branch lengths of *H. ammodendron*, the more assimilative the branches, confirming Corner (1949) second rule that there was a trade-off between branch size and number (Westoby and Wright, 2003).

There was no significant difference in BD and LAB between *H. ammodendron* and *E. przewalskii* in this study, but *H. ammodendron* had significantly lower DM than *E. przewalskii*. Therefore, the findings, *E. przewalskii* had a lower specific leaf area (SLA) than *H. ammodendron*, implying that evergreen plants (*E. przewalskii*) have lower SLA and photosynthetic efficiency, as well as a longer leaf lifespan (Liu and Liang, 2016). The DM of *H. ammodendron* was lower than that of the other three shrubs, which could be attributed to the fine structure of the assimilative branches. Previous research has shown that the water storage tissues in *H. ammodendron* assimilative branches are well developed, accounting for more than 50% of their radius, and that the assimilative branches of this succulent plant have a very high moisture content, possibly due to a large amount of water loss after drying, resulting in a significant reduction in DM (Tan et al., 2011; Zan and Zhuang, 2017; Wei et al., 2020). *E. przewalskii* is an evergreen plant that has been subjected to more environmental stress than the other three species. According to studies, the dry mass of evergreens is greater than that of other deciduous plants, implying that the leaf construction cost of evergreens is frequently greater than that of deciduous plants (Niklas, 1999; Cornelissen et al., 2003; Li et al., 2008; Zhu et al., 2011).

The NI of *H. ammodendron* and *H. persicum* was significantly higher than that of *C. mongolicum* and *E. przewalskii*, as shown in **Figure 3**. This difference was consistent with the individual size differences observed in four shrubs previously studied. As a result, we hypothesized that there might be a link between NI and plant individual size. Furthermore, studies have shown that the structural characteristics of leaves change with plant size, though some changes are not significant (Price et al., 2014; Huang et al., 2016; Chang et al., 2021). For leaves that initially grow between the internodes, there is a larger number of nodes per assimilative branch, which indicates that the shrub originally had more full-grown leaves. In addition, we found that the AL of *H. ammodendron* and *H. persicum* had significantly smaller values than that of *C. mongolicum* and *E. przewalskii*, and LAB and DM of *H. ammodendron* and *H. persicum* were slightly smaller than those of *C. mongolicum* and *E. przewalskii* at later growth stages. Therefore, we speculated that the length of assimilative branches developed at the earlier stages of plant growth may affect the total length of all assimilative branches, which may be related to the potential configuration of plants. For *C. mongolicum* and *H. ammodendron* with similar heights, studies have shown that the latter has the fractal dimension of the branches twice that of the former and also a more complex branch structure (Guo et al., 2015). From a biomechanical perspective, internode length, dry mass, and basal diameter may potentially play critical roles in plant resistance to bending and also in structural stability (Yang et al., 2016; Stubbs et al., 2022). Tall plants with complex branch structures not only bear greater water loss through transpiration and mechanical damage but also are more susceptible to strong wind interference. Therefore, by adjusting the length, dry mass, and basal diameter of assimilative branches, their ability to resist bending can be enhanced to some extent (Li et al., 2021).

In addition, it is undeniable that the differences in all traits of assimilative branches should not be ignored to a certain extent for the other appendages carried by assimilative branches (Li et al., 2009). Moreover, assimilative branches are not only the most important photosynthetic organ but also the basis of sexual reproduction. Since the construction and maintenance of tips, stipules, and other appendages undoubtedly require biomass allocation, the existence of these structures may affect the existence of assimilative branches and cause changes in them, just as the fruit structure affects the cross-species variation in seed yield (Henery and Westoby, 2001). However, it must be pointed out that the four shrubs can grow and develop normally in the arid environment due to their multiple channels and successful adaptation to drought, and any adaptation mode has its value (Zhang, 2002).

### Allometric relationships among functional traits of assimilative branches of desert shrubs

The allometric relationship among different functional traits is an ecological strategy for plants to promote resource utilization which reflects the ability of plants to coordinately adapt to environmental changes (Wu et al., 2021). The results of a goodness-of-fit test for all morphological traits of assimilative branches showed a good fit, and all traits showed a non-proportional increasing trend, indicating that the allocation ratio of resources among assimilating branch traits was different. Since DM, NI, and LAB of assimilative branches are the material basis of plant growth and reproduction, as well as the key factor for morphogenesis of photosynthetic organs, resources are more likely to be allocated to them, similar to the law of “diminishing returns” of investment supported by Niklas (Niklas et al., 2007). The allometric relationship among morphological traits of assimilative branches had specificity, indicating that different species have adapted to environmental conditions in different types of the morphological structure of their leaves (Yang et al., 2004; Guerra and Scremin-Dias, 2018). It might be due to differences in fine structures (palisade tissue, stomatal structure, etc.) of assimilative branches.

We found that the scaling exponent between all traits and the DM of assimilative branches of *H. persicum* also were higher or significantly higher than those of *H. ammodendron*, and there were no significant differences in the scaling exponent between NI, BD, and AL and between LAB, NI, and BD. The habitat heterogeneity and different ecological adaptation strategies of plants lead to differences in investment trade-off mechanisms, which directly or indirectly affect the growth, development, and reproduction of plants (Xu et al., 2014; Wu et al., 2021).

The scaling exponents between LAB, NI, AL, and DM, as well as between NI and LAB and NI and BD in assimilative branches of *C. mongolicum* were higher than those of the other three shrubs, while the scaling exponent between BD, DM, and LAB was the smallest. The results indicated that compared with the other three shrubs in *C. mongolicum*, more resources were allocated for the length and number of nodes of assimilative branches but less for their diameter. This may be related to the clonal growth of *C. mongolicum* (Li SY et al., 2013; Fan et al., 2016). Studies have reported that the assimilative branches of *C. mongolicum* show high phenotypic plasticity when exposed to heterogeneous environmental conditions such as wind erosion and sand burial (Balestri and Lardicci, 2013; Fan et al., 2016; Fan et al., 2018a; Fan et al., 2018b). Clonal plants acquire resources in the micro-environment through morphological changes and biomass allocation; for example, to escape from unfavorable habitat conditions as soon as possible and reduce their losses, they mainly establish the simplified structure and slow down their growth by developing thinner stolons, increasing the length of internodes, and reducing the number of branches (D’Hertefeldt and van der Putten, 1998; Zhang et al., 2022).

In addition, the allometric relationships between some traits of *E. przewalskii* were not significantly different from those of the other three plants. Among them, the assimilative branches of *E. przewalskii* and *H. ammodendron* had the same allometric relationship between their BD and LAB, which indicated that although there are quite phylogenetic and individual differences between them, they have experienced the same geological and historical events and the arid and seasonal temperate desert climate during their evolution (Xie et al., 2012) and the basal diameter and total length of their assimilative branches have a relatively consistent resource allocation rate.

However, *E. przewalskii*, an evergreen shrub, is better able to cope with environmental stress than the other three deciduous plants and prefers to allocate limited photosynthetic products for leaf thickening to resist harsh environments during the non-growing seasons and improve its stress resistance (Cornelissen et al., 2003; Wei et al., 2020). In addition, several reports have shown that evergreen plants take a long time to establish the supporting structures (e. g., basal diameter), which may decrease their photosynthetic efficiency so that they accumulate photosynthetic products by extending the lifespan of their leaves and increasing the length of time during which photosynthesis occurs in leaves (Westoby and Wright, 2003; Wright et al., 2004; Li et al., 2008; Choat et al., 2012).

Under the restriction of resources, natural selection develops the final geometry and structure of plant leaves in the most economical way. Only by constantly adjusting the proportion of distributed internal resources and ultimately establishing a morphological structure conducive to their growth can plants enhance their adaptation to adversity (Yang et al., 2004). As the important organ of the desert shrub, the complexity of morphology and internal structure of assimilative branches is closely related to the environment. During the long-term evolution, under the influence of systematic classification, genetic development, and environmental factors, each species has its unique biological characteristics and evolutionary adaptation process, and there are differences in the response and adaptation to the external environment; thus, different species have the same or different allometric relationships among their various traits (Liu et al., 2008). However, there are many similar adaptation strategies. Data obtained on the morphological and structural parameters of assimilating branches of four shrubs are limited, and therefore, more data need to be collected to verify the specific reasons for these similarities and differences.

## Conclusions

In conclusion, we discovered that the variation of NI and AL was greatest among species, whereas the variation of LAB, BD and DM was greater among individuals and assimilative branches. The characteristics of four desert shrubs’ assimilative branches differed. *C. mongolicum* and *E. przewalskii* have longer internodes and fewer nodes than *H. persicum* and *H. ammodendron*. *H. ammodendron* had the smallest dry mass and *H. persicum* had the smallest basal diameter. The reason could be that different plants have different functional requirements for different leaf forms. The scaling exponents between dry mass, length, basal diameter, and each assimilative branch trait differed between four shrub species, all of which were significantly less than one, indicating that the resource allocation ratio among assimilative branch traits varied. The allometric relationship between each trait-pair of assimilative branches differed significantly between four shrub species, which could be related to plant characteristics such as individual size and species specificity formed as a result of long-term adaptation to the desert environment. From the standpoint of the allometric growth relationship between traits, the findings of our study provide an in-depth understanding of the survival strategies and ecological functions of shrubs with assimilative branches that have adapted to the arid desert environment with high temperatures.

## Data availability statement

The original contributions presented in the study are included in the article/**Supplementary Material**. Further inquiries can be directed to the corresponding authors.

## Author contributions

YT and D-QZ: conceptualization, investigation, writing – review and editing, and preparation. H-HM: data analysis, writing – original draft. YT, B-FY, Y-GL and X-BZ: investigation, formal analysis, and methodology. Y-MZ and YT: conceptualization, resources, supervision, funding acquisition, project administration, and writing – review and editing. All authors contributed to the article and approved the submitted version.
