# Supplementary material for: Differences and allometric relationships among assimilative branch traits of four shrubs in Central Asia
Source: Front Plant Sci. 2022 Dec 13;13:1064504. doi: 10.3389/fpls.2022.1064504 (PMC9793409; doi:10.3389/fpls.2022.1064504)
Supplement: Supplementary file 1 [file Table_1.docx]

Supplementary Material

# Supplementary Tables

Table S1. Allometric relationships among LAB, NI, BD, AL and DM of four desert shrubs.

| Parameter | Species | *R*^2^ | *P* | Slope | 95% CI | Y-Intercept | 95% CI |
| --- | --- | --- | --- | --- | --- | --- | --- |
| Y = LAB  X = DM | HP | 0.936 | 0.001 | 0.700 b | 0.655,0.747 | 1.941a | 1.882, 2.000 |
|  | HA | 0.922 | 0.000 | 0.597 c | 0.554,0.642 | 1.901 | 1.838, 1.964 |
|  | CM | 0.952 | 0.000 | 0.832 a | 0.786,0.881 | 2.171 | 2.112, 2.230 |
|  | EP | 0.932 | 0.000 | 0.666 b | 0.622,0.713 | 1.881b | 1.828, 1.935 |
| Y = NI  X = DM | HP | 0.869 | 0.000 | 0.414 c | 0.377, 0.456 | 1.769b | 1.719, 1.819 |
|  | HA | 0.840 | 0.000 | 0.394 c | 0.355, 0.438 | 1.846a | 1.787, 1.906 |
|  | CM | 0.904 | 0.000 | 0.722 a | 0.665, 0.783 | 1.683 | 1.610, 1.756 |
|  | EP | 0.788 | 0.000 | 0.556 b | 0.493, 0.628 | 1.363 | 1.284, 1.442 |
| Y = BD  X = DM | HP | 0.859 | 0.000 | 0.258 a | 0.234, 0.285 | 0.368b | 0.336, 0.400 |
|  | HA | 0.764 | 0.000 | 0.219 b | 0.192, 0.248 | 0.438 | 0.398, 0.478 |
|  | CM | 0.633 | 0.000 | 0.151 c | 0.129, 0.177 | 0.291 | 0.261, 0.321 |
|  | EP | 0.807 | 0.000 | 0.267 a | 0.238, 0.300 | 0.448a | 0.412, 0.485 |
| Y = AL  X = DM | HP | 0.505 | 0.000 | 0.347 a | 0.289, 0.418 | 0.449 b | 0.367, 0.531 |
|  | HA | 0.715 | 0.000 | 0.273 b | 0.238, 0.314 | 0.293 | 0.238, 0.348 |
|  | CM | 0.252 | 0.000 | 0.419 a | 0.334, 0.525 | 0.908 a | 0.789, 1.027 |
|  | EP | 0.079 | 0.031 | 0.339 ab | 0.263, 0.436 | 0.815 a | 0.712, 0.917 |

Slope is the allometric scaling exponent, *R^2^* is the determination coefficient of the species, *P* is the allometric significance of the species.

Table S2. Allometric relationships among NI, BD, AL and LAB of four desert shrubs.

| Parameter | Species | *R*^2^ | *P* | Slope | 95% CI | Y-Intercept | 95% CI |
| --- | --- | --- | --- | --- | --- | --- | --- |
| *Y* = NI  X = LAB | HP | 0.830 | 0.000 | 0.593 b | 0.532,0.660 | 0.619 a | 0.549, 0.689 |
|  | HA | 0.928 | 0.000 | 0.661 b | 0.616,0.710 | 0.589 b | 0.540, 0.639 |
|  | CM | 0.854 | 0.000 | 0.867 a | 0.785,0.959 | -0.200 a | -0.302, -0.098 |
|  | EP | 0.807 | 0.000 | 0.836 a | 0.745,0.938 | -0.210 b | -0.318, -0.102 |
| *Y* = BD  *X* = LAB | HP | 0.825 | 0.000 | 0.369 a | 0.331, 0.412 | -0.349 b | -0.393, -0.305 |
|  | HA | 0.581 | 0.000 | 0.366 a | 0.309, 0.434 | -0.258 a | -0.324, -0.192 |
|  | CM | 0.476 | 0.000 | 0.181 b | 0.150, 0.219 | -0.102 | -0.143, -0.062 |
|  | EP | 0.680 | 0.000 | 0.401 a | 0.346, 0.465 | -0.306 a | -0.373, -0.240 |
| *Y* = AL  *X* = LAB | HP | 0.586 | 0.000 | 0.497 a | 0.420, 0.588 | -0.515 c | -0.606, -0.424 |
|  | HA | 0.758 | 0.000 | 0.458 a | 0.403, 0.521 | -0.578 d | -0.641, -0.515 |
|  | CM | 0.392 | 0.000 | 0.504 a | 0.411, 0.617 | -0.185 b | -0.306, -0.064 |
|  | EP | 0.092 | 0.019 | 0.506 a | 0.394, 0.649 | -0.140 a | -0.283, 0.003 |

Table S3. Allometric relationships between NI and BD of four desert shrubs.

| Parameter | Species | *R*^2^ | *P* | Slope | 95% CI | Y-Intercept | 95% CI |
| --- | --- | --- | --- | --- | --- | --- | --- |
| *Y* = NI  *X* = BD | HP | 0.622 | 0.000 | 1.606 c | 1.367, 1.886 | 1.179a | 1.158, 1.199 |
|  | HA | 0.553 | 0.000 | 1.805 bc | 1.516, 2.150 | 1.055b | 1.011, 1.099 |
|  | CM | 0.693 | 0.000 | 4.784 a | 4.138, 5.532 | 0.290 | 0.211, 0.368 |
|  | EP | 0.708 | 0.000 | 2.084b | 1.809, 2.401 | 0.428c | 0.384, 0.473 |
